# CARE COORDINATION, HEALTH OUTCOMES, AND HEALTHCARE UTILIZATION AMONG ADULTS WITH MULTIMORBIDITY

**DOI:** 10.1093/geroni/igac059.082

**Published:** 2022-12-20

**Authors:** Briana Mezuk, Weidi Qin, Linh Dang, Rodlescia Sneed

**Affiliations:** University of Michigan, School of Public Health, Ann Arbor, Michigan, United States; University of Michigan, Ann Arbor, Michigan, United States; University of Michigan, Ann Arbor, Michigan, United States; Wayne State University, Detroit, Michigan, United States

## Abstract

Care coordination is a vehicle for improving patient-provider and provider-provider communication to improve outcomes and reduce unnecessary healthcare utilization, particularly for adults with multimorbidity. However, the clinical effectiveness of coordination at the population level remains unknown. This study examined the association between experiences of care coordination with subsequent health and healthcare outcomes among US adults over age 50. The analytic sample (n=695) included respondents from the Health and Retirement Study who had at least two chronic conditions, completed an Experimental Module on Coordinated Care in 2016, and were re-interviewed in 2018. Three domains of care coordination were examined as predictors: perceptions of coordination; using tangible supports (e.g., seeing a care coordinator); and using technical supports (e.g., patient portal). A range of outcomes related to health (i.e., self-rated health, functioning, pain) and healthcare (i.e., medication adherence, visits, hospitalizations, care satisfaction) were assessed in 2018. Weighted linear and logistic regression models, adjusted for demographic and socioeconomic characteristics, were fit for each lagged outcome. Higher engagement with tangible supports was positively associated with subsequent hospitalization (OR: 1.08, 95%CI: 1.01-1.15), greater pain (OR: 1.11 , 95%CI: 1.03-1.20), and marginally worse self-rated health (B=-0.02, p< 0.063). Better perceptions of coordination were also positively associated with care satisfaction (B=0.03, p< 0.020). Care coordination was not associated with functioning, adherence, or number of medical visits. Findings indicate the salience of tangible support for coordination among older adults with multi-morbidity, and that positive perceptions of coordination contribute to healthcare satisfaction.

